# Multicenter retrospective analysis of 581 patients with primary intestinal non-hodgkin lymphoma from the Consortium for Improving Survival of Lymphoma (CISL)

**DOI:** 10.1186/1471-2407-11-321

**Published:** 2011-07-29

**Authors:** Seok Jin Kim, Chul Won Choi, Yeung-Chul Mun, Sung Yong Oh, Hye Jin Kang, Soon Il Lee, Jong Ho Won, Min Kyoung Kim, Jung Hye Kwon, Jin Seok Kim, Jae-Yong Kwak, Jung Mi Kwon, In Gyu Hwang, Hyo Jung Kim, Jae Hoon Lee, Sukjoong Oh, Keon Woo Park, Cheolwon Suh, Won Seog Kim

**Affiliations:** 1 Division of Hematology and Oncology, Department of Medicine, Samsung Medical Center, Sungkyunkwan University School of Medicine, Seoul, Korea; 2 Department of Internal Medicine, Korea University Guro Hospital, Korea University College of Medicine, Seoul, Korea; 3 Department of Internal Medicine, Ewha Womans University School of Medicine, Seoul, Korea; 4 Department of Internal Medicine, Dong-A University College of Medicine, Busan, Korea; 5 Department of Internal Medicine, Korea Cancer Center Hospital, Seoul, Korea; 6 Department of Internal Medicine, Dankook University College of Medicine, Cheonan, Korea; 7 Department of Internal Medicine, Soon Chun Hyang University Hospital, Seoul, Korea; 8 Department of Internal Medicine, Yeungnam University College of Medicine, Daegu, Korea; 9 Department of Hematology-Oncology, Kangdong Sacred Heart Hospital, Hallym University College of Medicine, Seoul, Korea; 10 Division of Hematology, Department of Internal Medicine, Yonsei University College of Medicine, Seoul, Korea; 11 Department of Internal Medicine, Chonbuk National University Medical School, Jeonju, Korea; 12 Department of Internal Medicine, Jeju University College of Medicine, Jeju, Korea; 13 Department of Medicine, Chung-Ang University College of Medicine, Seoul, South Korea; 14 Department of Internal Medicine, Hallym University Sacred Heart Hospital, Hallym University College of Medicine, Anyang, Korea; 15 Division of Hematology/Oncology, Gachon University Gil Hospital, Gachon University of Medcine and Science, Incheon, Korea; 16 Department of Internal Medicine, Kangbuk Samsung Hospital, Sungkyunkwan University, School of Medicine, Seoul, Korea; 17 Department of Oncology, Asan Medical Center, University of Ulsan College of Medicine, Seoul, Korea

**Keywords:** intestine, non-Hodgkin lymphoma, prognosis, histopathology

## Abstract

**Background:**

Primary intestinal non-Hodgkin lymphoma (NHL) is a heterogeneous disease with regard to anatomic and histologic distribution. Thus, analyses focusing on primary intestinal NHL with large number of patients are warranted.

**Methods:**

We retrospectively analyzed 581 patients from 16 hospitals in Korea for primary intestinal NHL in this retrospective analysis. We compared clinical features and treatment outcomes according to the anatomic site of involvement and histologic subtypes.

**Results:**

B-cell lymphoma (n = 504, 86.7%) was more frequent than T-cell lymphoma (n = 77, 13.3%). Diffuse large B-cell lymphoma (DLBCL) was the most common subtype (n = 386, 66.4%), and extranodal marginal zone B-cell lymphoma of mucosa-associated lymphoid tissue (MALT) was the second most common subtype (n = 61, 10.5%). B-cell lymphoma mainly presented as localized disease (Lugano stage I/II) while T-cell lymphomas involved multiple intestinal sites. Thus, T-cell lymphoma had more unfavourable characteristics such as advanced stage at diagnosis, and the 5-year overall survival (OS) rate was significantly lower than B-cell lymphoma (28% versus 71%, P < 0.001). B symptoms were relatively uncommon (20.7%), and bone marrow invasion was a rare event (7.4%). The ileocecal region was the most commonly involved site (39.8%), followed by the small (27.9%) and large intestines (21.5%). Patients underwent surgery showed better OS than patients did not (5-year OS rate 77% versus 57%, P < 0.001). However, this beneficial effect of surgery was only statistically significant in patients with B-cell lymphomas (P < 0.001) not in T-cell lymphomas (P = 0.460). The comparison of survival based on the anatomic site of involvement showed that ileocecal regions had a better 5-year overall survival rate (72%) than other sites in consistent with that ileocecal region had higher proportion of patients with DLBCL who underwent surgery. Age > 60 years, performance status ≥ 2, elevated serum lactate dehydrogenase, Lugano stage IV, presence of B symptoms, and T-cell phenotype were independent prognostic factors for survival.

**Conclusions:**

The survival of patients with ileocecal region involvement was better than that of patients with involvement at other sites, which might be related to histologic distribution, the proportion of tumor stage, and need for surgical resection.

## Background

The gastrointestinal tract is the most commonly involved extranodal location of non-Hodgkin lymphoma (NHL) [1,2]. The intestines are the second most common site of involvement following the stomach, and account for 30 to 40% of primary gastrointestinal lymphomas [1-3]. However, information regarding primary intestinal NHL is relatively scarce because the majority of previous studies focused on gastric lymphoma [1,3,4]. The limited number of studies about primary intestinal NHL analyzed relatively small numbers of patients [5-12]. Another problem is that the classification of the pathology differs depending on the study period, as the majority of studies were retrospective analyses [1,4-6,9,13-15]. The use of old histologic classifications, such as the Kiel classification, makes comparisons among reported results difficult [1,5,6,9,11].

The ambiguity of anatomic classification is another obstacle to the analysis of primary intestinal NHL. Diseases involving the intestines are dichotomized into small and large intestinal diseases depending on the affected anatomic site. However, primary intestinal NHL most commonly involves the ileocecal region, probably due to the high proportion of lymphoid tissue [4,6,16]. Because the ileocecal region includes the area from the distal ileum to the cecum, it is often difficult to designate the ileocecal region as part of the small or large intestine. Thus, the designation for this region differs among studies, as some considered it part of the small or large intestine [1,9,10], and others distinguished it from the small and large intestine entirely [4,17]. Therefore, the estimated incidence rates of small and large intestinal lymphoma also varied among studies [4,17].

Due to this heterogeneity with regard to anatomic and histologic distribution of primary intestinal NHL, studies focusing on primary intestinal NHL in large patient samples using current pathologic classifications are warranted to understand this disease entity. Therefore, we analyzed data from Korean patients with primary intestinal NHL in the present multicenter retrospective study. We distinguished the ileocecal region from the small and large intestine for the purposes of classification. We analyzed the histologic distribution of primary intestinal NHL, and compared the clinical features and survival outcomes of patients.

## Methods

### Patients and tumor localization

Patients who presented with predominant intestinal lesions were defined as primary intestinal NHL according to the definition for primary gastrointestinal tract lymphoma proposed in previous reports [18,19]. Pathological diagnoses were made according to the Revised European-American Lymphoma (REAL) classification or the World Health Organization (WHO) classification depending on the time of diagnosis. Cases with ambiguous histologic diagnosis or insufficient data regarding the pathology were excluded from this analysis. Tumor locations were determined using imaging findings, such as computerized tomography (CT), or surgical findings if surgical resection was performed. Small intestinal lymphomas were considered to be lymphomas between the duodenum and the ileum, while large intestinal lymphomas were considered to be lymphomas between the ascending colon and the rectum. The ileocecal region was defined as the area between the distal ileum to the cecum.

### Clinical data

Investigators affiliated with the Consortium for Improving Survival of Lymphoma (CISL) reviewed medical records and gathered clinical data for patients diagnosed with primary intestinal NHL between 1993 and 2010. Data included patient demographics and clinical features at diagnosis including stage, Eastern Cooperative Oncology Group (ECOG) performance status, serum lactate dehydrogenase (LDH), international prognostic index (IPI), histologic subtypes, the presence of B symptoms, and tumor location. Not all patients underwent colonoscopy for diagnosis because substantial number of patients underwent surgery to remove primary mass as diagnostic and therapeutic purpose. Thus, the specimen for pathologic diagnosis was obtained from biopsy under colonoscopy or surgically removed primary mass. Few patients underwent other specialized diagnostic techniques such as capsule endoscopy and double balloon endoscopy. All patients underwent imaging studies for staging work-up, including chest and abdomen-pelvis CT scans. The results of positron emission tomography (PET)/CT scan were not included in this study because a limited number of patients underwent PET/CT scan for their staging work-up. Patients were staged according to the Lugano staging system for gastrointestinal lymphomas as previously reported [20,21]. Stage I is defined as disease confined to the intestine, stage II is defined as disease extending to local (II-1) or distant (II-2) nodes, stage II-E is defined as disease involving adjacent organs or tissues, and stage IV is defined as disseminated extranodal involvement or concomitant supradiaphragmatic lymph node involvement. The IPI risk was calculated from five parameters including age, performance status, serum LDH, number of extranodal involvement and Lugano stage. Clinical manifestation related with intestinal lesions such as intestinal obstruction, bleeding and perforation were analyzed because other symptoms were not specific to intestinal lesions. Data regarding treatments and outcomes include type of primary treatment, treatment response, and survival status. Response was defined according to WHO criteria [22]. The institutional review board of each participating center approved this retrospective analysis, which was a part of the larger CISL study registered at http://www.clinicaltrials.gov (#NCT01043302).

### Statistical analysis

The Fisher's exact test was applied to assess the association between categorical variables, and the Kruskal-Wallis test was used to compare mean values. Overall survival (OS) was calculated from the date of diagnosis to the date of the final follow-up or death from any cause. Progression-free survival (PFS) was calculated from the date of diagnosis to the date of disease progression, relapse, or death from any cause. Survival was estimated using Kaplan-Meier curves and compared by the log-rank test. The Cox proportional hazard regression model was used in multivariate analyses to identify prognostic factors. Two-sided *P *values < 0.05 were considered significant.

## Results

### Primary site of involvement

We enrolled 581 patients from 16 hospitals in Korea for primary intestinal NHL in this retrospective analysis. 361 patients (62.1%) underwent colonoscopy for diagnostic purpose while 220 patients were diagnosed after surgery. Among patients undergoing colonoscopy, 334 patients were pathologically diagnosed as NHL whereas 27 patients were not diagnosed by colonoscopic biopsy. These 27 patients were diagnosed after surgical resection of primary intestinal mass. The majority of patients involved had single lesions in the intestines (89.2%). The ileocecal region was the most commonly involved site (n = 231, 39.8%, Table 1). Multiple intestinal involvement cases included the combined involvement of small and large intestines, and the involvement of two or more lesions within the small or large intestines (n = 63, 10.8%). Multiple intestinal involvements was significantly more frequent in T-cell lymphoma. The jejunal involvement was also more common in T-cell than B-cell lymphomas (15.6% versus 4.4%), thus, T-cell lymphomas showed more frequent involvement of the small intestine (P = 0.02). B-cell lymphomas accounted for the majority of ileocecal region lymphoma (n = 221, 95.7%).

**Table 1:** Anatomic distribution of primary intestinal NHL

Primary site	Total cases (n = 581)	B-cell lymphoma (n = 504)	T-cell lymphoma (n = 77)	P value*
Small intestine				
Duodenum	31 (5.3)	25 (5.0)	6 (7.8)	0.02
Jejunum	34 (5.9)	22 (4.4)	12 (15.6)	
Ileum	97 (16.7)	84 (16.7)	13 (16.9)	
Ileocecal region	231 (39.8)	221 (43.8)	10 (13.0)	< 0.001
Large intestine				
Ascending/transverse colon	87 (15.0)	70 (13.9)	17 (22.1)	0.14
Descending/sigmoid colon	12 (2.1)	11 (2.2)	1 (1.3)	
Rectum	26 (4.5)	25 (5.0)	1 (1.3)	
Multiple intestinal Involvement	63 (10.8)	46 (9.1)	17 (22.1)	0.002

NA: not applicable

*Fisher's exact test was used to assess the association between immunophenotype and the primary site in small and large intestine.

### Characteristics of patients

The median age of the patients was 56 years (range: 15-92 years), and the male to female ratio was 1.71:1. Most patients had good performance status (≤ ECOG grade 0/1, 84.3%) and localized disease (Lugano stage I/II 71.1%). Thus, the IPI risks in our patients were mainly low or low intermediate (75.4%). B symptoms were relatively uncommon (20.7%), and bone marrow invasion was a rare event in primary intestinal NHL (7.4%, Table 2). Clinical presentations associated with intestinal obstruction such as intussusceptions were found in 96 patients (16.5%), and all these patients underwent emergent surgery. The frequency of bleeding (n = 13, 2.2%) and perforation (n = 25, 4.3%) was relatively lower than obstruction. Among the cases with perforation, 10 cases occurred during chemotherapy. When the characteristics of patients were compared according to the primary site of involvement, there were no significant differences. Only patients with multiple intestinal involvements were more likely to show high or high-intermediate IPI risk (Table 2).

**Table 2:** Comparison of clinical features based on primary site of involvement

Characteristics		Total cases (n = 581)	Small intestine (n = 162)	Ileocecal region (n = 231)	Large intestine (n = 125)	Multiple intestinal involvement (n = 63)	P value
Age (years)	≤ 60	356 (61.3)	100 (61.7)	146 (63.2)	77 (61.6)	33 (52.4)	0.479
	> 60	225 (38.7)	62 (38.3)	85 (36.8)	48 (38.4)	30 (47.6)	
Sex	Male	367 (63.2)	108 (66.7)	146 (63.2)	73 (58.4)	40 (63.5)	0.557
	Female	214 (36.8)	54 (33.3)	85 (36.8)	52 (41.6)	23 (36.5)	
Performance status	ECOG 0/1	490 (84.3)	135 (83.9)	197 (85.3)	104 (83.2)	54 (85.7)	0.942
	ECOG ≥ 2	90 (15.5)	26 (16.0)	34 (14.7)	21 (16.8)	9 (14.3)	
	Missing	1 (0.2)	1 (0.1)				
Serum LDH level	Normal	355 (61.1)	92 (56.8)	152 (65.8)	77 (61.6)	34 (54.0)	0.086
	Increased	210 (36.1)	67 (41.4)	71 (30.7)	43 (34.4)	29 (46.0)	
	Missing	16 (2.8)	3 (1.8)	8 (3.5)	5 (4.0)		
B symptoms	Absent	459 (79.0)	125 (77.2)	185 (80.1)	103 (82.4)	46 (73.0)	0.441
	Present	120 (20.7)	36 (22.2)	45 (19.5)	22 (17.6)	17 (27.0)	
	Missing	2 (0.3)	1 (0.6)	1 (0.4)			
Intestinal symptoms	Obstruction	96 (16.5)	25 (15.4)	47 (20.3)	14 (11.2)	10 (15.9)	0.267
	Bleeding	13 (2.2)	2 (1.2)	8 (3.5)	3 (2.4)	0 (0.0)	
	Perforation	25 (4.3)	8 (4.9)	13 (5.6)	3 (2.4)	1 (1.6)	
Extranodal involvement	< 2	417 (71.8)	105 (64.8)	179 (77.5)	103 (82.4)	30 (47.6)	< 0.001
	≥ 2	155 (26.7)	55 (34.0)	47 (20.3)	21 (16.8)	32 (50.8)	
	Missing	9 (1.5)	2 (1.2)	5 (2.2)	1 (1.0)	1 (1.6)	
IPI	L/LI	277/151 (75.4)	76/40 (71.6)	129/48 (76.6)	59/38 (77.6)	13/25 (60.3)	< 0.001
	HI/H	87/53 (22.4)	24/20 (27.2)	33/15 (20.8)	16/8 (19.2)	14/10 (38.1)	
	Missing	13 (2.2)	2 (1.2)	6 (2.6)	4 (3.2)	1 (1.6)	
Lugano stage	I/II	139/264 (71.1)	37/73 (67.9)	54/126 (77.9)	43/54 (77.6)	5/21 (41.3)	< 0.001
	IV	168 (28.9)	52 (32.1)	51 (22.1)	28 (22.4)	37 (58.7)	
BM invasion	Absent	494 (85.0)	131 (80.9)	199 (86.1)	111 (88.8)	53 (84.1)	0.300
	Present	43 (7.4)	17 (10.5)	13 (5.6)	6 (4.8)	7 (11.1)	
	ND	44 (7.6)	14 (8.6)	19 (8.2)	8 (6.4)	3 (4.8)	
Immunophenotype	B-cell	504 (86.7)	131 (80.9)	221 (95.7)	106 (84.8)	46 (73.0)	< 0.001
	T-cell	77 (13.3)	31 (19.1)	10 (4.3)	19 (15.2)	17 (27.0)	

ECOG: Eastern Cooperative Oncology Group; LDH: lactate dehydrogenase; IPI: International Prognostic Index; L: low; LI: low-intermediate; HI: high-intermediate; H: high; BM: bone marrow; ND: not done

### Histological distribution

Diffuse large B-cell lymphoma (DLBCL) was the most common subtype (n = 386, 66.4%), and extranodal marginal zone B- cell lymphoma of mucosa-associated lymphoid tissue (MALT) was the second most common subtype (n = 61, 10.5%). Burkitt lymphoma (BL, n = 31, 5.3%), mantle cell lymphoma (MCL, n = 19, 3.3%) and follicular lymphoma (FL, n = 7, 1.2%) together comprised only a minor fraction of intestinal NHL cases. The proportion of T-cell lymphomas was relatively small (n = 77, 13.3%) including three subtypes: peripheral T-cell lymphoma, unspecified (PTCL-U, n = 34, 5.9%), enteropathy-associated T-cell lymphoma (EATL, n = 25, 4.3%) and extranodal NK/T cell lymphoma (ENKTL, n = 18, 3.1%).

### Comparison of histologic subtypes

The median age of MCL (60 years, Table 3) was the highest while BL, PTCL-U, and ENKTL had younger median ages (P = 0.002, Table 3). The majority of DLBCL and MALT cases presented as localized disease, while other subtypes more frequently presented as Lugano stage IV. The proportion of high/high-intermediate IPI risk patients was greater in the group with BL (Table 3). T-cell lymphoma showed more frequent occurrence of B symptoms (> 35%). The ileocecal region was the most common primary site of involvement in DLBCL. The large intestine was the most common primary site in MALT, thus, eleven cases of MALT occurred in the rectum (11/61, 18.0%). Multiple intestinal involvements such as multicentric involvement were more frequent in MCL (57.9%), and the pattern of intestinal involvement in MCL was peculiar. Thus, multi-centric involvement through entire colon like intestinal polyposis was frequently found in colonoscopy.

**Table 3:** Comparison of clinical features based on histological subtype

Characteristics	DLBCL No. (%)	MALT No. (%)	BL No. (%)	MCL No. (%)	FL No. (%)	PTCL-U No. (%)	EATL No. (%)	ENKTL No. (%)	P value
Number of cases	386	61	31	19	7	34	25	18	
Median age (range)	56 (15-92)	55 (15-80)	47 (15-78)	60 (42-78)	52 (39-81)	49 (15-78)	51 (23-75)	47 (32-72)	0.002
Age > 60, %	160 (41.5)	22 (36.1)	7 (22.6)	10 (52.6)	3 (42.9)	11 (32.4)	7 (28.0)	5 (27.8)	0.246
Male, %	240 (62.2)	32 (52.5)	25 (80.6)	13 (68.4)	4 (57.1)	24 (70.6)	17 (68.0)	12 (66.7)	0.273
Performance status ≥ 2, %	60 (15.6)	6 (9.8)	5 (16.1)	0 (0.0)	1 (14.3)	9 (26.5)	6 (24.0)	3 (16.7)	0.218
Lugano stage IV, %	94 (24.4)	7 (11.5)	17 (54.8)	15 (78.9)	3 (42.9)	15 (44.1)	8 (32.0)	9 (50.0)	< 0.001
Increased serum LDH, %	150 (39.9)	5 (8.5)	23 (76.7)	4 (21.1)	1 (14.3)	12 (36.4)	8 (34.8)	7 (38.9)	< 0.001
Presence of B symptoms, %	75 (19.5)	7 (11.5)	7 (22.6)	3 (15.8)	1 (14.3)	12 (35.3)	9 (36.0)	6 (35.3)	0.048
Extranodal involvement ≥ 2, %	93 (24.3)	3 (5.4)	18 (58.1)	9 (47.4)	2 (28.6)	10 (29.4)	10 (41.7)	10 (55.6)	< 0.001
IPI HI/H, %	93 (24.5)	5 (8.9)	15 (48.4)	7 (36.8)	1 (14.3)	9 (26.5)	6 (26.1)	4 (22.2)	0.008
Bone marrow invasion, %	20 (5.2)	4 (6.6)	7 (22.6)	5 (26.3)	0 (0.0)	3 (8.8)	3 (12.0)	1 (5.6)	< 0.001
Intestinal obstruction, %	69 (17.8)	7 (11.5)	6 (19.4)	0 (0.0)	2 (28.5)	4 (11.8)	4 (16.0)	4 (22.2)	0.398
Bleeding, %	8 (2.0)	2 (3.3)	1 (3.2)	0 (0.0)	0 (0.0)	1 (2.9)	1 (4.0)	0 (0.0)	0.964
Perforation, %	19 (4.9)	1 (1.6)	0 (0.0)	0 (0.0)	0 (0.0)	2 (5.9)	3 (12.0)	0 (0.0)	0.194
Small intestine, %	104 (26.9)	14 (23.0)	11 (35.5)	0 (0.0)	2 (28.6)	13 (38.2)	10 (40.0)	8 (44.4)	< 0.001
Ileocecal region, %	187 (48.4)	19 (31.1)	9 (29.0)	3 (15.8)	3 (42.9)	7 (20.6)	2 (8.0)	1 (5.6)	< 0.001
Large intestine, %	73 (18.9)	21 (34.4)	5 (16.1)	5 (26.3)	2 (28.6)	5 (14.7)	10 (40.0)	4 (22.2)	< 0.001
Multiple intestinal lesions, %	22 (5.7)	7 (11.5)	6 (19.4)	11 (57.9)	0 (0.0)	9 (26.5)	3 (12.0)	5 (27.8)	< 0.001

LDH: lactate dehydrogenase; IPI: International Prognostic Index; HI: high-intermediate; H: high; DLBCL: diffuse large B-cell lymphoma; MALT: extranodal marginal zone B-cell lymphoma; BL: Burkitt lymphoma; PTCL-U: peripheral T-cell lymphoma, unspecified; EATL: enteropathy-associated T-cell lymphoma; MCL: mantle cell lymphoma; ENKTL: extranodal natural killer/T-cell lymphoma; FL: follicular lymphoma

### Treatments and outcomes

Chemotherapy was the predominant treatment in patients with primary intestinal NHL regardless of the involved site. Thus, the majority of patients received chemotherapy as a curative treatment (n = 521, 89.7%, Table 4). Various chemotherapy regimens were used, although CHOP or rituximab-CHOP was the main regimen for lymphoma, therefore, comparisons of outcomes based on chemotherapy regimens were not performed. Surgical resection was performed in 289 patients (49.7%) for diagnostic and/or therapeutic purposes as mentioned earlier. Among patients diagnosed by colonoscopy, some patients underwent surgery to remove primary mass of intestine. The ileocecal region was the most common site of surgery (64.1%). Radiotherapy was used less frequently than chemotherapy and surgery. However, radiotherapy was used frequently in patients with MALT (n = 13, 21.3%) compared to other subtypes, while approximately half of all patients with MALT received chemotherapy due to indolent clinical courses (n = 30, 49.2%, Table 5). The overall response rates of DLBCL, BL and MCL were greater than 80% while PTCL-U, EATL and ENKTL showed around 50% of the overall response rate. Consistent with these findings, the proportion of relapse or progression was higher in PTCL-U, EATL and ENKTL, and this fact lead to a higher number of deaths than in B-cell subtypes. Among B-cell lymphomas, relapse or progression was more frequent in MCL and FL, even though they showed a relatively high overall response rate.

**Table 4:** Comparison of treatments and outcomes based on primary site

Characteristics	Total cases (n = 581)	Small intestine (n = 162)	Ileocecal region (n = 231)	Large intestine (n = 125)	Multiple intestinal involvement (n = 63)
Treatment*					
Chemotherapy	521 (89.7%)	143 (88.3%)	213 (92.2%)	105 (84.0%)	60 (95.2%)
Surgical resection	289 (49.7%)	74 (45.7%)	148 (64.1%)	49 (39.2%)	18 (28.6%)
Radiotherapy	56 (9.6%)	21 (13.0%)	18 (7.8%)	13 (10.4%)	4 (6.3%)
Response					
Complete response	360 (62.0%)	94 (58.0%)	164 (71.0%)	71 (57.0%)	31 (49.0%)
Partial response	62 (10.7%)	16 (9.9%)	16 (6.9%)	19 (15.0%)	11 (18.0%)
Outcome					
Relapse or Progression	199 (34.3%)	57 (35.2%)	65 (28.1%)	50 (40.0%)	27 (42.9%)
Dead	152 (26.2%)	44 (27.2%)	47 (20.3%)	36 (28.8%)	25 (39.7%)
Survival					
Median OS	Not reached	Not reached	Not reached	140 months	61 months
5-year OS	67%	65%	72%	67%	55%
Median PFS	88 months	55 months	115 months	66 months	28 months
5-year PFS	53%	50%	62%	50%	37%

*Some patients were treated with combined modality such as surgery plus chemotherapy. Thus, the sum of number of each treatment is larger than total number of patients.

**Table 5:** Comparison of treatments and outcomes based on histologic subtypes

Characteristics	DLBCL No. (%)	MALT No. (%)	BL No. (%)	MCL No. (%)	FL No. (%)	PTCL-U No. (%)	EATL No. (%)	ENKTL No. (%)
Treatment*								
Chemotherapy	368 (95.3)	30 (49.2)	29 (93.5)	19 (100.0)	5 (71.4)	30 (88.2)	23 (92.0)	17 (94.4)
Surgical resection	223 (57.8)	25 (41.0)	9 (29.0)	1 (5.3)	3 (42.9)	9 (26.5)	12 (48.0)	7 (38.9)
Radiotherapy	32 (8.3)	13 (21.3)	2 (6.5)	1 (5.3)	2 (28.6)	4 (11.8)	1 (4.0)	1 (5.6)
Response								
Complete response	264 (68.4)	36 (59.0)	22 (71.0)	11 (57.9)	4 (57.1)	11 (32.4)	7 (28.0)	5 (27.8)
Partial response	36 (9.3)	5 (8.2)	5 (16.1)	5 (26.3)	0 (0.0)	4 (11.8)	4 (16.0)	3 (16.7)
Outcome								
Relapse or Progression	112 (29.0)	14 (23.0)	11 (35.5)	8 (42.1)	4 (57.1)	19 (55.9)	18 (72.0)	13 (73.2)
Dead	87 (22.5)	8 (13.1)	7 (22.6)	6 (31.6)	2 (28.6)	18 (52.9)	15 (60.0)	9 (50.0)
Survival								
Median OS	Not reached	Not reached	Not reached	46 months	54 months	35 months	8.6 months	7 months
5-year OS	72%	88%	76%	39%	42%	23%	35%	45%
Median PFS	Not reached	115 months	Not reached	31 months	16 months	10 months	4.2 months	4 months
5-year PFS	58%	80%	60%	0%	22%	17%	23%	21%

*Some patients were treated with combined modality such as surgery plus chemotherapy. Thus, the sum of number of each treatment is larger than total number of patients.

DLBCL: diffuse large B-cell lymphoma; MALT: extranodal marginal zone B-cell lymphoma; BL: Burkitt lymphoma; PTCL-U: peripheral T-cell lymphoma, unspecified; EATL: enteropathy-associated T-cell lymphoma; MCL: mantle cell lymphoma; ENKTL: extranodal natural killer/T-cell lymphoma; FL: follicular lymphoma

### Survival and prognostic factors

**Figure 1:**
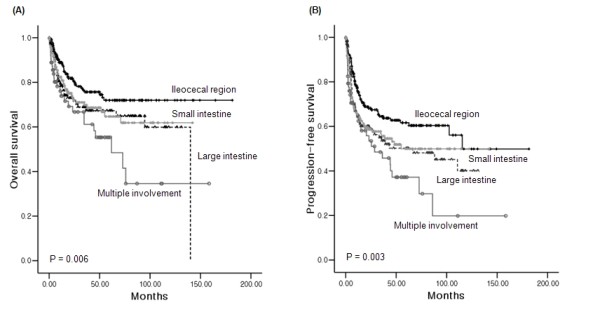
**Comparison of survival curves based on the site of involvement**. (A, B) Overall and progression-free survival curves according to primary site of involvement. Patients with ileocecal region involvement had better survival outcomes than patients with involvement of the small and large intestines. The outcomes of patients with multiple intestinal involvement were significantly worse (P < 0.01).

**Figure 2:**
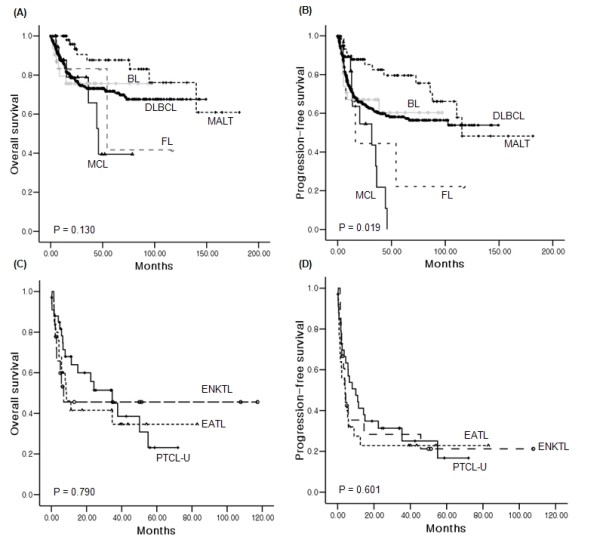
**Comparison of survival curves based on the histologic subtypes**. (A, B) Overall and progression-free survival curves according to subtype of B-cell lymphoma. MALT lymphoma showed better OS than other subtypes, while BL and DLBCL showed similar OS curves to each other. (C, D) Overall and progression-free survival curves according to subtype of T-cell lymphoma. There were no significant differences among PTCL-U, EATL, and ENKTL.

**Figure 3:**
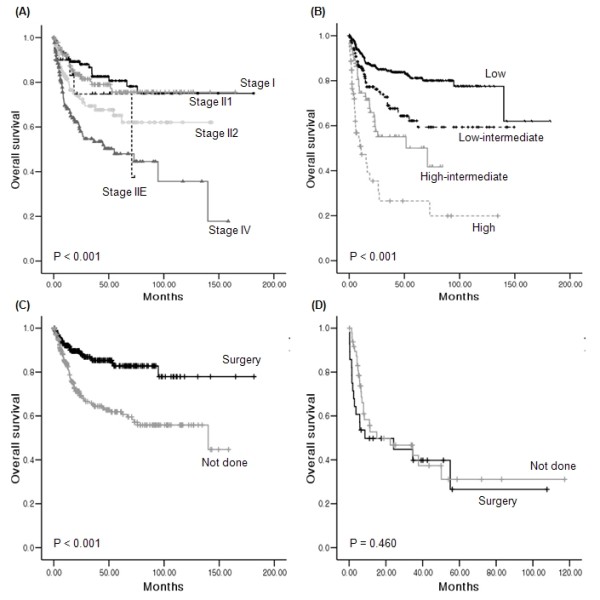
**Comparison of survival curves based on the clinical characteristics**. (A) Lugano stage II2 and IV cases had significantly worse OS, while there were no significant differences in OS between stage I and II1. (B) IPI was significantly associated with OS. (C) In B-cell lymphoma, patients who underwent surgical resectioning had better OS than patients that did not. (D) Surgical resections failed to lead to survival differences in T-cell lymphoma.

The 5-year OS and PFS rates of ileocecal NHL were 72% and 62%, respectively, while the small and large intestines showed similar survival rates (Figure 1). The 5-year OS rate of B-cell lymphoma was significantly better than that of T-cell lymphoma (71% versus 28%, P < 0.001). The comparison of OS in all subtypes of B-cell lymphoma did not show a significant difference (Figure 2A, P = 0.130). However, when the OS of MALT was compared with that of DLBCL and MCL, the OS of MALT was significantly better than DLBCL and MCL (P = 0.021 and 0.001, respectively). There were no significant differences of OS among PTCL-U, EATL, and ENKTL, although the median OS (34.3 months) of PTCL-U was longer than that of ENKTL (8.6 months) and EATL (7.0 months, Figure 2B). The PFS of MCL and FL was shorter than other subtypes of B-cell NHL (Figure 2C). However, the PFS of three T-cell subtypes showed similar outcomes (Figure 2D). Patients with Lugano stage II2 and IV disease had significantly worse OS than stage I and II1 (Figure 3A). Other parameters affecting the IPI score, such as age, ECOG performance status, serum LDH, and the number of extranodal involvements were also significantly associated with OS (data not shown). Thus, the IPI showed a clear association with OS (P < 0.001, Figure 3B). Patients who underwent surgical resection had better OS than patients who did not undergo surgery (5-year OS rate 77% versus 57%, P < 0.001). However, the survival benefit associated with surgical resection was significant only in B-cell lymphomas and not in T-cell lymphomas (Figure 3C, D). Multivariate analyses with these parameters for OS showed that age > 60 years, poor performance status, elevated serum LDH, Lugano stage IV, presence of B symptoms, and T-cell phenotype were independent predictive indicators for poor OS (Table 6).

**Table 6:** Multivariate analysis of prognostic factors

Characteristics	P value	Hazard ratio	95% Confidence Interval	
			
			Lower limit	Upper limit
Age > 60	< 0.001	1.945	1.379	2.743
Performance status ≥ 2	< 0.001	2.072	1.384	3.101
Elevated serum LDH	0.002	1.776	1.233	2.558
Extranodal involvement ≥ 2	0.579	0.892	0.596	1.335
Lugano stage IV	0.001	1.248	1.090	1.429
Multiple intestinal involvement	0.357	1.076	0.920	1.259
Immunophenotype T-cell	< 0.001	3.645	2.454	5.416
B symptoms	0.028	1.530	1.046	2.237
Surgical resection not done	0.281	1.235	0.842	1.811

## Discussion

Primary intestinal NHL accounts for a major proportion of cases of extranodal lymphoma. Although its prognosis is poor compared to gastric lymphoma, there are few studies analyzing the clinical features and survival outcomes of primary intestinal NHL according to primary site of involvement and histologic subtype. In this study, we analyzed data for 581 patients, making ours the largest sample among studies investigating primary gastrointestinal lymphoma. The clinical features of our study were similar to those described in previous studies, and revealed that primary intestinal NHL occurs more frequently in male patients and predominantly presents as a localized disease (Table 7).

**Table 7:** Summary of published results of prospective and retrospective studies

References	Study type	Time period	Nationality	number	Location	M/F	B/T cell	Stage I/II vs. III/IV	B-cell	T-cell
d'Amore et al [1]	Retrospective	1983-1991	Denmark	109	SI/LI	76/33	93/16	56 vs. 48	High grade (51)	PTCL (10)
									Intermediate grade (18)	ALCL (6)
								Unknown (3)	Low grade (21)	
Koch et al [4]	Retrospective	1992-1996	Germany	58	SI/LI	40/18	48/10	52 vs. 6	High grade (39)	T-cell (10)
									Low grade (4)	
									BL/LBL (5)	
Kohno et al [6]	Retrospective	1981-2000	Japan	143	SI/LI	109/34	122/21	Not described	Large cell (84), BL (16)	PTCL (15)
									MALT (10), MCL (7)	ENKTL (2)
									FL (4)	ALCL (2)
Daum et al [16]	Prospective	1995-1999	Germany	56	SI/LI	25/31	21/35	42 vs. 14	DLBCL (18)	EATL (28)
									MALT (2), FL (1)	Unknown (7)
Yin et al [12]	Retrospective	1996-2005	China	34	SI	22/12	27/7	22 vs. 12	DLBCL (24)	Unknown (7)
									MALT (3)	
Kako et al [10]	Retrospective	1990-2007	Japan	23	SI	16/7	20/3	11 vs. 12	DLBCL (15), FL (1)	EATL (2)
									MCL (1), MALT (2)	ALCL (1)
									Unknown (1)	
Li et al [9]	Retrospective	1992-2003	China	40	SI/LI	26/14	38/2	28 vs. 12	DLBCL (17)	PTCL (1)
									MALT (20)	Unknown (1)
									Unknown (1)	
Wong et al [8]	Retrospective	1989-1999	Singapore	14	LI	13/1	14/0	5 vs. 9	DLBCL (8), MCL (4)	
									BL (2)	

SI: small intestine; LI: large intestine; DLBCL: diffuse large B-cell lymphoma; MALT: extranodal marginal zone B-cell lymphoma; BL: Burkitt lymphoma; PTCL-U: peripheral T-cell lymphoma, unspecified; EATL: enteropathy-associated T-cell lymphoma; MCL: mantle cell lymphoma; ENKTL: extranodal natural killer/T-cell lymphoma; FL: follicular lymphoma; LBL: lymphoblastic lymphoma

The incidence of B-cell lymphoma was much that of higher than T-cell lymphoma, and DLBCL was the main subtype. This is consistent with the observation that the majority of gastrointestinal tract NHL is of B-cell origin, including DLBCL and MALT lymphoma [2,3,8,23]. However, the proportion of DLBCL (n = 386, 66.4%) was significantly higher than MALT (n = 64, 10.6%) in our study. This is different from gastric lymphoma, in which MALT lymphoma accounts for approximately 40% of all cases [15,23]. This high frequency of DLBCL might be associated with the worse prognosis of intestinal lymphoma compared to gastric lymphoma [1,3,15,24]. The relatively higher incidence of T-cell lymphoma may be another cause of the poor prognosis for intestinal NHL. Our study showed the occurrence of three subtypes of T-cell lymphoma including PTCL-U, EATL and ENKTL with a frequency of 13.2%. Although the proportion of T-cell lymphomas varied according to the type of study and number of patients [5,9,16], our proportion was comparable to previous studies with a relatively large number of patients [1,3,4]. Patients with T-cell lymphomas more frequently presented with advanced disease and constitutional B symptoms, and their overall response rate to treatment was inferior to that of B-cell lymphomas. This resulted in significantly worse survival outcomes for T-cell lymphoma compared to B-cell lymphoma in our study, which is consistent with previous results [7,16]. The comparison of survival outcomes based on subtypes of NHL demonstrated that MCL did not show a survival curve plateau. This reflects MCL has higher risk of relapse resulting in worse OS and PFS than other subtypes (Figure 2) in consistent with previous results [25-27]. The 5-year OS of PTCL-U in our study was inferior to previously reported 5-year OS of nodal PTCL-U, suggesting a poor prognosis for intestinal T-cell lymphoma [28].

The ileocecal region was the most common site of involvement, accounting for approximately 40% of primary sites in this study (Table 1). However, this region was mainly affected by B-cell lymphomas (95.7%). The frequent occurrence of B-cell lymphomas in the ileocecal region was associated with high proportions of DLBCL (Table 2). T-cell lymphomas were extremely rare in the ileocecal region (4.3%), while involvement of the jejunum was more common in T-cell lymphomas (12.5%) than in B-cell (3.6%). This relatively high incidence of T-cell lymphomas in the small intestine, especially the jejunum, was also noted in previous studies [3,4,6]. Like previous studies reporting high proportions of MALT lymphoma in the duodenum and rectum in East Asian samples [6], the high proportion of B-cell lymphoma in the duodenum and rectum in this study was also associated with frequent occurrence of MALT lymphoma.

A comparison of survival outcomes based on primary site of involvement revealed that involvement of the ileocecal region was associated with better survival rates than involvement of the small and large intestine. Patients with multiple intestinal involvements had the worst survival outcomes. A previous study reported that the overall survival of ileocecal lymphoma was similar to that of gastric lymphoma and superior to that of small intestinal lymphoma [4]. There are several possible explanations for the superior survival outcomes of patients with involvement in the ileocecal region. First, T-cell lymphoma rarely occurs in the ileocecal region compared to the small and large intestine. Thus, the proportion of T-cell lymphoma in our study (4.3%) was similar to that of a previous study reporting 4% in the ileocecal region [4]. Second, lymphomas in the ileocecal region often presented with complications, such as obstructions requiring surgical intervention. Thus, more than 50% of patients with lymphoma in the ileocecal region underwent immediate surgery [1,4,17,29,30]. Our study also showed that the percentage of patients who underwent surgery in the ileocecal region (64.1%) was significantly higher than the percentage of patients who required surgery in the small and large intestines (45.7% and 39.2%, respectively, Table 4). Previous studies reported that primary surgical treatment had a favourable influence on the prognosis of intestinal lymphoma, especially for localized disease [7,31]. Thus, the fact that many of our patients received surgery might explain the better survival of patients with ileocecal lymphoma in our study as compared to other studies.

The optimal treatment strategy for intestinal lymphoma is still unclear. Although conservative treatment is preferred to surgery in localized gastric lymphomas, the same is not true for intestinal lymphomas because surgery in combination with chemotherapy has proven superior to any other treatment combination [1,5]. In a previous study, we compared the outcomes of surgery followed by chemotherapy, and chemotherapy alone in intestinal DLBCL, and found that surgery followed by chemotherapy led to better survival outcomes [32]. Consistent with these findings, surgical resection was associated with survival benefits in patients with B-cell lymphoma in the present study (P < 0.001, Figure 3C). Considering the fact that more than 90% of patients received chemotherapy, this result may be interpreted to reflect a survival advantage of surgery plus chemotherapy. However, the survival benefit was not observed in patients with T-cell lymphoma (P = 0.460, Figure 3D), possibly due to the high proportion of Lugano stage IV cases in our sample. Thus, need for surgery failed to show independent prognostic value in the multivariate analysis for OS (Table 6). The results of our multivariate analysis demonstrated that age, performance status, serum LDH, Lugano stage, B symptoms, and T-cell immunophenotype were all independently prognostic for OS in patients with intestinal NHL.

Although this is the largest series of primary intestinal NHL, our study has some limitations. First, patients included in this analysis were not consecutively diagnosed because of its retrospective study in nature. Second, we could not provide the results of PET/CT scan because PET/CT scan was not widely used before 2006 in Korea.

## Conclusions

In summary, we determined clinical features and outcomes of patients with primary intestinal NHL. The survival of patients with ileocecal region involvement was better than that of patients with involvement at other sites, which might be related to histologic distribution, the proportion of tumor stage, and need for surgical resection. Factors associated with the IPI score and T-cell immunophenotype were shown to be prognostic in this disease entity. Surgical resection may provide survival benefits to patients with localized B-cell intestinal NHL.

## Competing interests

The authors declare that they have no competing interests.

## Authors' contributions

SJK participated in the design of the study and review of clinical data, and drafted the manuscript. HJK, JSK, SYO, CWC, SIL, KWP, JHW, MKK, JHK, YCM, JYK, JMK, IGH, HJK, JP, and SO recorded the clinical data. CS and WSK participated in the coordination of the study, and revised the manuscript. All authors read and approved the final manuscript.

## Pre-publication history

The pre-publication history for this paper can be accessed here:

http://www.biomedcentral.com/1471-2407/11/321/prepub

## References

[B1] d'Amore F Brincker H Gronbaek K Thorling K Pedersen M Jensen MK Andersen E Pedersen NT Mortensen LS Non-Hodgkin's lymphoma of the gastrointestinal tract: a population-based analysis of incidence, geographic distribution, clinicopathologic presentation features, and prognosis. Danish Lymphoma Study Group J Clin Oncol 1994 12 8 1673 1684 8040680 10.1200/JCO.1994.12.8.1673

[B2] Ko YH Kim CW Park CS Jang HK Lee SS Kim SH Ree HJ Lee JD Kim SW Huh JR REAL classification of malignant lymphomas in the Republic of Korea: incidence of recently recognized entities and changes in clinicopathologic features. Hematolymphoreticular Study Group of the Korean Society of Pathologists. Revised European-American lymphoma Cancer 1998 83 4 806 812 9708949

[B3] Nakamura S Matsumoto T Iida M Yao T Tsuneyoshi M Primary gastrointestinal lymphoma in Japan: a clinicopathologic analysis of 455 patients with special reference to its time trends Cancer 2003 97 10 2462 2473 10.1002/cncr.11415 12733145

[B4] Koch P del Valle F Berdel WE Willich NA Reers B Hiddemann W Grothaus-Pinke B Reinartz G Brockmann J Temmesfeld A Primary gastrointestinal non-Hodgkin's lymphoma: I. Anatomic and histologic distribution, clinical features, and survival data of 371 patients registered in the German Multicenter Study GIT NHL 01/92 J Clin Oncol 2001 19 18 3861 3873 11559724 10.1200/JCO.2001.19.18.3861

[B5] Zinzani PL Magagnoli M Pagliani G Bendandi M Gherlinzoni F Merla E Salvucci M Tura S Primary intestinal lymphoma: clinical and therapeutic features of 32 patients Haematologica 1997 82 3 305 308 9234576

[B6] Kohno S Ohshima K Yoneda S Kodama T Shirakusa T Kikuchi M Clinicopathological analysis of 143 primary malignant lymphomas in the small and large intestines based on the new WHO classification Histopathology 2003 43 2 135 143 10.1046/j.1365-2559.2003.01659.x 12877728

[B7] Lee J Kim WS Kim K Ko YH Kim JJ Kim YH Chun HK Lee WY Park JO Jung CW Intestinal lymphoma: exploration of the prognostic factors and the optimal treatment Leuk Lymphoma 2004 45 2 339 344 10.1080/10428190310001593111 15101721

[B8] Wong MT Eu KW Primary colorectal lymphomas Colorectal Dis 2006 8 7 586 591 10.1111/j.1463-1318.2006.01021.x 16919111

[B9] Li B Shi YK He XH Zou SM Zhou SY Dong M Yang JL Liu P Xue LY Primary non-Hodgkin lymphomas in the small and large intestine: clinicopathological characteristics and management of 40 patients Int J Hematol 2008 10.1007/s12185-008-0068-5 18409078

[B10] Kako S Oshima K Sato M Terasako K Okuda S Nakasone H Yamazaki R Tanaka Y Tanihara A Kawamura Y Clinical outcome in patients with small intestinal non-Hodgkin lymphoma Leuk Lymphoma 2009 1 7 10.1080/10428190903147629 19672778

[B11] Kim YH Lee JH Yang SK Kim TI Kim JS Kim HJ Kim JI Kim SW Kim JO Jung IK Primary colon lymphoma in Korea: a KASID (Korean Association for the Study of Intestinal Diseases) Study Dig Dis Sci 2005 50 12 2243 2247 10.1007/s10620-005-3041-7 16416168

[B12] Yin L Chen CQ Peng CH Chen GM Zhou HJ Han BS Li HW Primary small-bowel non-Hodgkin's lymphoma: a study of clinical features, pathology, management and prognosis J Int Med Res 2007 35 3 406 415 17593870 10.1177/147323000703500316

[B13] Amer MH el-Akkad S Gastrointestinal lymphoma in adults: clinical features and management of 300 cases Gastroenterology 1994 106 4 846 858 8143991 10.1016/0016-5085(94)90742-0

[B14] Domizio P Owen RA Shepherd NA Talbot IC Norton AJ Primary lymphoma of the small intestine. A clinicopathological study of 119 cases Am J Surg Pathol 1993 17 5 429 442 10.1097/00000478-199305000-00001 8470758

[B15] Koch P del Valle F Berdel WE Willich NA Reers B Hiddemann W Grothaus-Pinke B Reinartz G Brockmann J Temmesfeld A Primary gastrointestinal non-Hodgkin's lymphoma: II. Combined surgical and conservative or conservative management only in localized gastric lymphoma--results of the prospective German Multicenter Study GIT NHL 01/92 J Clin Oncol 2001 19 18 3874 3883 11559725 10.1200/JCO.2001.19.18.3874

[B16] Daum S Ullrich R Heise W Dederke B Foss HD Stein H Thiel E Zeitz M Riecken EO Intestinal non-Hodgkin's lymphoma: a multicenter prospective clinical study from the German Study Group on Intestinal non-Hodgkin's Lymphoma J Clin Oncol 2003 21 14 2740 2746 10.1200/JCO.2003.06.026 12860953

[B17] Gurney KA Cartwright RA Gilman EA Descriptive epidemiology of gastrointestinal non-Hodgkin's lymphoma in a population-based registry Br J Cancer 1999 79 11-12 1929 1934 10206316 10.1038/sj.bjc.6690307 PMC2362786

[B18] Lewin KJ Ranchod M Dorfman RF Lymphomas of the gastrointestinal tract: a study of 117 cases presenting with gastrointestinal disease Cancer 1978 42 2 693 707 10.1002/1097-0142(197808)42:2<693::AID-CNCR2820420241>3.0.CO;2-J 354774

[B19] Isaacson PG Gastrointestinal lymphoma Hum Pathol 1994 25 10 1020 1029 10.1016/0046-8177(94)90060-4 7927306

[B20] Rohatiner A d'Amore F Coiffier B Crowther D Gospodarowicz M Isaacson P Lister TA Norton A Salem P Shipp M Report on a workshop convened to discuss the pathological and staging classifications of gastrointestinal tract lymphoma Ann Oncol 1994 5 5 397 400 8075046 10.1093/oxfordjournals.annonc.a058869

[B21] Cortelazzo S Rossi A Oldani E Motta T Giardini R Zinzani PL Zucca E Gomez H Ferreri AJ Pinotti G The modified International Prognostic Index can predict the outcome of localized primary intestinal lymphoma of both extranodal marginal zone B-cell and diffuse large B-cell histologies Br J Haematol 2002 118 1 218 228 10.1046/j.1365-2141.2002.03613.x 12100151

[B22] Miller AB Hoogstraten B Staquet M Winkler A Reporting results of cancer treatment Cancer 1981 47 1 207 214 10.1002/1097-0142(19810101)47:1<207::AID-CNCR2820470134>3.0.CO;2-6 7459811

[B23] Koch P Probst A Berdel WE Willich NA Reinartz G Brockmann J Liersch R del Valle F Clasen H Hirt C Treatment results in localized primary gastric lymphoma: data of patients registered within the German multicenter study (GIT NHL 02/96) J Clin Oncol 2005 23 28 7050 7059 10.1200/JCO.2005.04.031 16129843

[B24] Nakamura S Matsumoto T Takeshita M Kurahara K Yao T Tsuneyoshi M Iida M Fujishima M A clinicopathologic study of primary small intestine lymphoma: prognostic significance of mucosa-associated lymphoid tissue-derived lymphoma Cancer 2000 88 2 286 294 10.1002/(SICI)1097-0142(20000115)88:2<286::AID-CNCR7>3.0.CO;2-Z 10640959

[B25] Zucca E Roggero E Pinotti G Pedrinis E Cappella C Venco A Cavalli F Patterns of survival in mantle cell lymphoma Ann Oncol 1995 6 3 257 262 7612491 10.1093/oxfordjournals.annonc.a059155

[B26] Pinotti G Zucca E Roggero E Pascarella A Bertoni F Savio A Savio E Capella C Pedrinis E Saletti P Clinical features, treatment and outcome in a series of 93 patients with low-grade gastric MALT lymphoma Leuk Lymphoma 1997 26 5-6 527 537 9389360 10.3109/10428199709050889

[B27] Zucca E Conconi A Pedrinis E Cortelazzo S Motta T Gospodarowicz MK Patterson BJ Ferreri AJ Ponzoni M Devizzi L Nongastric marginal zone B-cell lymphoma of mucosa-associated lymphoid tissue Blood 2003 101 7 2489 2495 10.1182/blood-2002-04-1279 12456507

[B28] Sonnen R Schmidt WP Muller-Hermelink HK Schmitz N The International Prognostic Index determines the outcome of patients with nodal mature T-cell lymphomas Br J Haematol 2005 129 3 366 372 10.1111/j.1365-2141.2005.05478.x 15842660

[B29] Ruskone-Fourmestraux A Aegerter P Delmer A Brousse N Galian A Rambaud JC Primary digestive tract lymphoma: a prospective multicentric study of 91 patients. Groupe d'Etude des Lymphomes Digestifs Gastroenterology 1993 105 6 1662 1671 8253342 10.1016/0016-5085(93)91061-l

[B30] Turowski GA Basson MD Primary malignant lymphoma of the intestine Am J Surg 1995 169 4 433 441 10.1016/S0002-9610(99)80193-7 7694986

[B31] Lee J Kim WS Kim K Ahn JS Jung CW Lim HY Kang WK Park K Ko YH Kim YH Prospective clinical study of surgical resection followed by CHOP in localized intestinal diffuse large B cell lymphoma Leuk Res 2007 31 3 359 364 10.1016/j.leukres.2006.06.018 16930692

[B32] Kim SJ Kang HJ Kim JS Oh SY Choi CW Lee SI Won JH Kim MK Kwon JH Mun YC Comparison of treatment strategies for patients with intestinal diffuse large B-cell lymphoma: surgical resection followed by chemotherapy versus chemotherapy alone Blood 2011 117 6 1958 1965 10.1182/blood-2010-06-288480 21148334

